# To explore immune synergistic function of Quercetin in inhibiting breast cancer cells

**DOI:** 10.1186/s12935-021-02345-5

**Published:** 2021-11-27

**Authors:** Dan Qiu, Xianxin Yan, Xinqin Xiao, Guijuan Zhang, Yanqiu Wang, Jingyu Cao, Ruirui Ma, Shouyi Hong, Min Ma

**Affiliations:** 1 School of Traditional Chinese Medicine, Jinan University, No. 601, West Huangpu Avenue, Guangzhou, 510632 Guangdong China; 2 School of Nursing, Jinan University, No. 601, West Huangpu Avenue, Guangzhou, 510632 Guangdong China; 3 The First Affiliated Hospital of Jinan University, Jinan University, No. 601, West Huangpu Avenue, Guangzhou, 510632 Guangdong China

**Keywords:** Que, Breast cancer, Breast precancerous lesion, γδ T cell, JAK/STAT1 signaling pathway

## Abstract

**Background:**

The precancerous disease of breast cancer is an inevitable stage in the tumorigenesis and development of breast neoplasms. Quercetin (Que) has shown great potential in breast cancer treatment by inhibiting cell proliferation and regulating T cell function. γδ T cells are a class of nontraditional T cells that have long attracted attention due to their potential in immunotherapy. In this study, we revealed the immunomodulatory function of Que through regulation of the JAK/STAT1 signaling pathway, which was followed by the synergistic killing of breast cancer cells.

**Methods:**

In the experimental design, we first screened target genes with or without Que treatment, and we intersected the Que target with the disease target by functional enrichment analysis. Second, MCF-10A, MCF-10AT, MCF-7 and MDA-MB-231 breast cancer cell lines were treated with Que for 0 h, 24 h and 48 h. Then, we observed the expression of its subsets by coculturing Que and γδ T cells and coculturing Que and γδ T cells with breast tumor cells to investigate their synergistic killing effect on tumor cells. Finally, Western blotting was used to reveal the changes in proteins related to the JAK/STAT1 signaling pathway after Que treatment in MCF-10AT and MCF-7 cells for 48 h.

**Results:**

The pathway affected by Que treatment was the JAK/STAT1 signaling pathway and was associated with precancerous breast cancer, as shown by network pharmacology analysis. Que induced apoptosis of MCF-10AT, MCF-7 and MDA-MB-231 cells in a time- and concentration-dependent manner (*P* < *0.05*). Most importantly, Que promoted the differentiation of γδ T cells into the Vδ2 T cell subpopulation. The best ratio of effector cells to target cells (E/T) was 10:1, the killing percentages of γδ T cells against MCF-10A, MCF-10AT, MCF-7, and MDA-MB-231 were 61.44 ± 4.70, 55.52 ± 3.10, 53.94 ± 2.74, and 53.28 ± 1.73 (*P* = *0.114, P* = *0.486,* and *P* = *0.343*, respectively), and the strongest killing effect on precancerous breast cancer cells and breast cancer cells was found when the Que concentration was 5 μM and the E/T ratio was 10:1 (64.94 ± 3.61, 64.96 ± 5.45, 55.59 ± 5.98, and 59.04 ± 5.67, respectively). In addition, our results showed that Que increased the protein levels of IFNγ-R, p-JAK2 and p-STAT1 while decreasing the protein levels of PD-L1 (*P* < *0.0001*).

**Conclusions:**

In conclusion, Que plays a synergistic role in killing breast cancer cells and promoting apoptosis by regulating the expression of IFNγ-R, p-JAK2, p-STAT1 and PD-L1 in the JAK/STAT1 signaling pathway and promoting the regulation of γδ T cells. Que may be a potential drug for the prevention of precancerous breast cancer and adjuvant treatment of breast cancer.

## Background

Precancerous lesions of breast cancer refer to some breast diseases that are not malignant tumors but have an increased possibility of developing into malignant tumors. The World Health Organization (WHO) defines such lesions as precancerous because they are more than 20% likely to transform from nonneoplastic lesions to tumors [1]. The next stage in the continuous development of precancerous breast lesions is breast tumors. The incidence of breast cancer is increasing year by year and now has the highest incidence of malignant tumors in the world [2, 3]. Selective estrogen receptor modulators and aromatase inhibitors have become regular treatments for precancerous breast cancer lesions. However, these two drugs target estrogen receptors, resulting in endocrine disorders and a series of side effects. Researchers have been attempting to scientifically classify breast cancer according to its histopathological and molecular properties to better understand its pathological diagnostic value [4], although the theoretical community considers it an intractable task. Endocrine therapy, surgery and radiochemotherapy are the main therapies for breast cancer, but these traditional treatment methods have low remission rates and high recurrence rates and are not the best choice for patients. Combination therapy involves targeted therapy and/or immunotherapeutic therapy combined with the traditional therapies mentioned above. This new therapeutic strategy has made rapid progress in recent years with significant positive effects, providing new hope for patients with breast carcinomas.

The study and development of food with anticancer effects is an important link in the field of antitumor therapy. Quercetin (Que; 3, 3’, 4’, 5’, 5,7-Pc-ntabyolroxyflavone) is a flavonoid that is widely found in wild fields, mainly in the form of rutin, quercetin, hypericin and so on. Que can be obtained by an acid hydrolysis reaction [5]. It is widely distributed in the leaves, flowers and fruits of fruits, vegetables and Chinese herbal medicine and has multiple biological activities and pharmacological effects. A direct link has been shown between dietary acid uptake and breast cancer risk, indicating that a low acid load dietary style may reduce breast cancer risk. It has been suggested that potential dietary genes interact with hormonal mechanisms, susceptibility genes, polymorphisms, and epigenetic regulatory mechanisms [6]. Therefore, an increasing number of researchers have begun to pay attention to dietary substances enriched in flavonoids such as Que. Current studies have shown that the in vitro effects of Que are anti-inflammatory, antitumor, antiplatelet aggregation, protection of endothelial cells, antioxidation, inhibition of T cell activation and so on [5, 7]. Previously, we found that Que can be effectively used to prevent and treat breast cancer through its estrogen and antiestrogen effects. Que reverses drug resistance and chemotherapy sensitization, inhibits cell growth and induces apoptosis, inhibits tumor cell invasion and transformation, and inhibits tumor angiogenesis [8, 9]. It has the potential to become an alternative or complementary drug for the treatment of breast cancer. First, Que is a native Chinese medicine monomer with estrogenic activity that can activate ERβ in human breast carcinoma cells (T47D) to produce antiestrogen effects, thereby affecting the proliferation of mammary cancer cells. Animal experiments have shown that Que reduces the risk of breast cancer [10]. Second, Que reverses the multidrug resistance (MDR) of tumors [11], effectively improving the effect of chemotherapy. Correlation studies found that Que can regulate the imbalance of Th1/Th2 cell subsets, induce the immune response, and play a normal role in immune regulation [12]. In animal experiments, the combined use of Que and doxorubicin effectively inhibited tumors in a breast cancer (4T1) mouse model, reduced cytotoxicity and improved therapeutic effects [13]. Moreover, Que can effectively inhibit the proliferation of tumor cells and even induce apoptosis by blocking the division cycle, interfering with the signal transduction of stem cells [14, 15], and regulating the expression of tumor-related genes and proteins [16]. In addition, the failure to effectively control tumor invasion and metastasis is the main reason for recurrence and death in breast cancer patients and is closely related to matrix metalloproteinases (MMPs). Que can inhibit the proliferation and invasion of breast cancer cells by downregulating the expression of MMP9 mRNA and protein [17]. In a mouse model of colorectal cancer, Que inhibited the survival and metastasis ability of colon 26 (CT26) cells by regulating MMPs and tissue inhibitors of metalloproteinases (TIMPs). Thereby, Que could inhibit the metastasis of cancer cells to the lung, which confirmed that Que may be an effective treatment for metastatic colorectal cancer [18]. Currently, the high cost of treating cancer and the low success rate of most conventional treatments has led to an urgent need for cost-effective prevention and treatment methods. In this context, it is naturally the most appropriate direction to study the interaction between diet and cancer. Therefore, this study aims to elucidate the key properties of Que on breast cancer precancerous lesions, apoptosis of breast cancer cells or regulation of immune cells. These properties are considered antitumor properties that can improve the therapeutic effect of breast cancer.

γδ T cells are innate immune cells, accounting for 0.5 ~ 5% of all peripheral T lymphocytes. γδ T cells are mainly densely distributed in the mucosal epithelium and belong to a special class of immune cells involved in acquired immunity and natural immunity. Its subgroups are usually divided into Vδ1 T cells and Vδ2 T cells [19]. γδ T cells play an immune-regulatory role in the immune response to many infectious and immune diseases and are known as the first line of defense against infection, autoimmune disease and cancer [20]. Some studies have confirmed that γδ T cells play an active role in the antitumor immune response of nasopharyngeal carcinoma, colon cancer, renal cancer, and nonsmall cell lung cancer [21–23]. γδ T cells isolated from breast cancer, lung cancer, ovarian cancer, colon cancer and other patients were found to effectively kill tumor cells or primary tumor cells when interleukin-2 (IL-2) was amplified in vitro [24]. This indicates that γδ T cells have strong antitumor activity. The presence of tumor-infiltrating γδ T cells in tumors is considered an important indicator of the recurrence and poor survival of breast cancer patients [25]. γδ T cell treatment has good prospects in tumor immunotherapy applications, and phase I/II clinical trials are underway [22]. Based on previous findings, our research team found that γδ T subsets, dominated by Vδ2 T subsets, had a significant killing effect on precancerous breast cancer cells. However, γδ T cells have complex biological activities, and γδ T cells in peripheral blood may also have functional subsets with different functions. Further study on γδ T cells can lead to the better application of γδ T cells for the adoptive immunotherapy of breast cancer and predict the prognosis of patients with precancerous breast cancer lesions.

The JAK-STAT1 signaling pathway is involved in the process of tumor cell recognition and tumor-driven immune escape [26]. STAT1 (signal transducer and activator of transcription 1) can promote apoptosis and inhibit the growth and differentiation of cells in the JAK/STAT1 pathway [27]. Specifically, in breast cancer, loss of STAT1 in the breast epithelium has been associated with neurodriven tumorigenesis and the development of spontaneous breast tumors in BALB/C mice, which are associated with loss of epithelial interferon regulatory factor (IRF1) and impaired T cell infiltration and killing capacity [28]. The coinhibitory signal of programmed death receptor 1 (PD-1)/programmed cell death 1 ligand 1 (PD-L1) comediated T cell activation inhibits the killing function of T cells. Tumor cells can effectively avoid the recognition, killing and clearance of the immune system. The PD-1/PD-L1 interaction plays a negative regulatory role in the human immune response [29]. PD-1 can maintain immune tolerance in the normal immune system. In the case of viral infection or tumors, PD-1 reduces the proliferation and activation of T cells by binding to its ligand, resulting in a weakened immune response [30]. At this point, the immune surveillance mechanism of the immune system on tumor cells fails, and immune escape occurs. In the JAK-STAT1 pathway, the transcription factor STAT1 in tumor cells can regulate the expression of PD-L1 and is positively correlated with the expression of PD-L1 [31]. In addition, we observed that PD-L1 binds to PD-1 receptors on activated T cells, which leads to a weakening of antitumor immunity by inhibiting T cell activation signals [32]. Therefore, reducing the expression of PD-L1 and/or decreasing its combination with PD-1 allows T cells to function normally, which plays an important role in the treatment of breast cancer by inhibiting the progression of precancerous lesions to malignant carcinoma.

In this study, we investigated the effect of Que on γδ T immune cells or its inhibitory effect on breast cancer cells and explored the immunomodulatory function of Que through the JAK/STAT1 signaling pathway and the underlying mechanisms of its synergistic killing of breast cancer cells.

## Materials and methods

### Que target gene identification

**Fig. 1:**
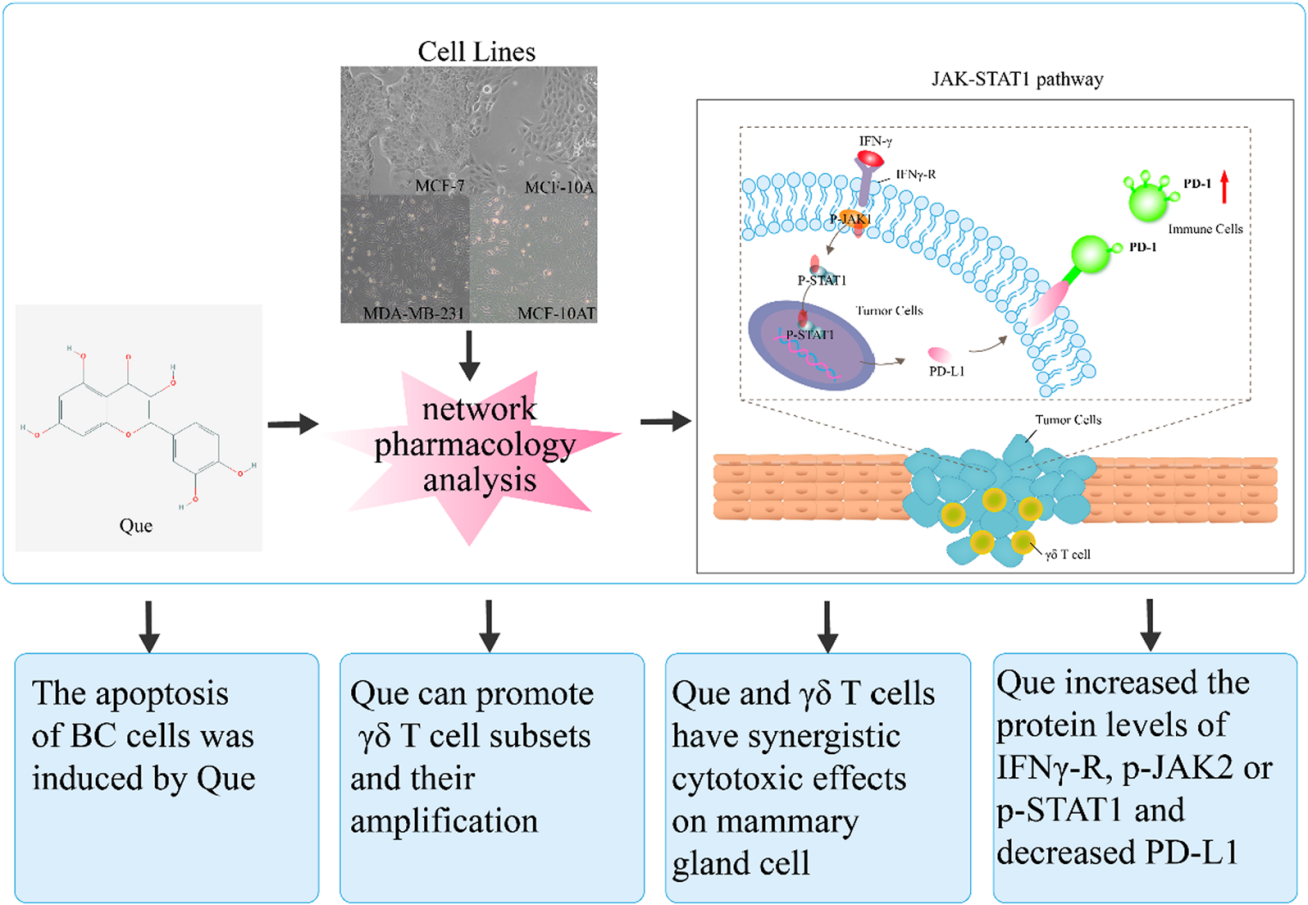
Schematic diagram of the study. Potential target genes were predicted and disease target genes were screened according to the structure of Que. Flow cytometry was used to detect the apoptotic effect of Que on breast tumor cells and IC_50_ was obtained by MTT method. PBMCs were isolated from peripheral blood of normal subjects by density gradient centrifugation. In vitro amplification was performed using anti-human TCR γδ monoclonal antibody and Que intervention. Flow cytometry was used to detect the changes of γδ T cell subsets. Que and γδ T cells were co-cultured with breast tumor cells, and the specific killing of γδ T cells on breast tumor cells was determined by flow cytometry. Finally, western blot was used to detect the expression of potentially related JAK/STAT1 signaling pathway proteins between Que and prebreast cancer

According to the structure of Que, the potential target genes of Que were predicted using the PharmMapper database, TCMSP database and Swiss Target Prediction database (Fig. 1).

### Prediction of precancerous lesions of breast cancer-related que target genes by a system pharmacology approach

Genes previously associated with breast cancer lesions were searched in the GeneCards database (https://www.genecards.org/) with “precancerous lesions” as the key word to obtain disease target information.

### GO and KEGG enrichment analysis

The target of Que was intersected with the disease target, and protein–protein interaction (PPI) analysis and GO and KEGG enrichment analysis were performed using the STRING database. PPI data were imported into Cytoscape 3.6.1 for visualization.

### Samples collection

After obtaining the consent of participants, peripheral blood samples were obtained from healthy controls (HCs), and peripheral blood mononuclear cells (PBMCs) were separated by Ficoll-Hyperaque gradient centrifugation. All the above procedures were carried out according to the guiding principles of the Medical Ethics Committee of Guangdong Provincial Health Bureau, and the ethics involved were examined and approved by the Ethics Committee of the First Affiliated Hospital of Jinan University in Guangdong Province.

### Cell culture

MCF-10A (a nontumorigenic epithelial cell line), MCF-10AT (breast cancer precancerous cells) and MCF-7 and MDA-MB-231 (human breast cancer cells) cells (American Type Culture Collection ATCC, USA) were maintained as monolayer cultures (25 cm^2^ plastic culture flasks) in F12/DMEM and DMEM (1x) medium with 10% fetal bovine serum (Gibco South America) and Donor Equine Serum (HyClone, USA), Penstrep at 37 °C in an incubator with an atmosphere containing 5% CO_2_. The cells were cultured every 2 days.

### Que was cocultured with γδ T cells

Peripheral blood of sterile anticoagulant normal subjects was collected and separated with lymphocyte separation solution to obtain peripheral blood mononuclear cells. Then, the cells were resuspended in RPMI 1640 complete medium (containing 500 μL double antibody + 10% fetal bovine serum) and coated in a 24-well culture plate with 1 μL (1 μg/mL) solid phase anti-human TCR γδ monoclonal antibody. Stimulated at 37 ℃ and 5%CO_2_ for 3 days. Starting on the fourth day, the solution was changed by centrifugation every other day, the supernatant was removed, and IL-2 (10 ng/mL) was added. Que working solution with concentrations of 0, 2.5, 5 and 10 μM was added to the culture medium (constant volume to 1 mL system). The culture was again placed at 37 ℃ and 5% CO_2_. γδ T cells were cultured for 16 days, Que was cultured for 13 days, and γδ T cell subsets were detected.

### MTT assay

The IC_50_ value of Que was detected by the MTT method. In short, MCF-10AT, MCF-7 and MDA-MB-231 cells were seeded in 96-well plates for 48 h. MTT solution (Sigma) was incubated at 37 ℃ for 4 h. A total of 140 μL of dimethyl sulfoxide (Sigma) working solution was added to each well and shaken on a low-speed shaker for 10 min to completely dissolve the crystal. The IC_50_ value of Que was determined by absorbance at 490 nm (Biotek, USA).

### Apoptosis assay

The apoptosis rates were verified by Annexin V staining. As negative controls, untreated cells were used, and cells treated with staurosporine (eBioscience, USA), which was used to induce apoptosis, were used as positive controls. Cells were collected, the number of cells was approximately 3*10^5^/mL, the cells were washed and centrifuged with PBS, and the supernatant was discarded. Then, 195 μL of binding buffer with 5 μL of annexin V-FITC (eBioscience, USA) was incubated for 15 min at room temperature. The cells were resuspended with 190 μL binding buffer and 10 μL of PI (20 µg/mL) were added to the machine and then the cells were immediately placed in the machine (Cytoflex S, Beckman Coulter, USA) to detect cell apoptosis, and data were analyzed by Flowjo software (Flowjo LLC, USA) (annexin −/PI−, (early apoptotic cells (annexin + /PI−, (late apoptotic cells (annexin + /PI +), and necrotic cells (annexin −/PI +).

### Flow cytometry

PBMCs were isolated from HC samples and then incubated with the following antibodies: CD3-FITC (clone SK7), TCR γδ-PE (clone IP26), Vδ1- PE-Cy7 (clone TS8.2), Vδ2-PerCP (clone B6), PE-Cy7 isotype control (clone P3.6.2.8.1), PE isotype control (clone eBM2a), FITC isotype control (clone MOPC-21), and PerCP isotype control (clone MOPC-21) (Biolegend, San Diego, USA; BD, Biosciences, San Jose, USA; Abcam Cambridge, UK). PBMCs were stained with different combinations of anti-human TCR γδ, Vδ1, Vδ2, and CD3 antibodies at room temperature in a dark room for 20 min. A total of 30,000–50,000 CD3\+ T cells were collected with a Cytoflex S flow cytometer (Beckman Coulter, USA), and data were analyzed by FlowJo software (FlowJo LLC, USA).

### Coculture of γδ T cells and MCF-10A, MCF-10AT, MCF-7, MDA-MB-231 cell proliferation treatment and CFDA SE (CFSE) assay

Subconfluent (80%) monolayers of MCF-10A, MCF-10AT, MCF-7, and MDA-MB-231 cells were trypsinized (Invitrogen, USA) and adjusted to 2 × 10^6^ cells/mL. One milliliter of resuspended related cell lines was removed and placed in a 1.5 mL centrifuge tube, and 0.4 μL CFSE (concentration of 5 mM) was added to a final sample concentration of 2 μM. Then, the trypsinized cells were preincubated with γδ T cells diluted in RPMI 1640 medium with 10% fetal bovine serum (Ausgenex, Australia) and Penstrep (Life Technologies, USA) at 37 °C for 4–5 h in a humidified atmosphere containing 5% CO_2_. After this period of coculture with γδ T cells, the cells were removed and stained with 0.5 μL PI, and the killing efficiency of γδ T cells was immediately detected by Cytoflex S (Beckman Coulter, USA).

### Western blot analysis

Total protein was extracted according to the protein extraction kit instructions. Protein concentration (Beyotime, China) was determined by the BCA method. After electrophoresis, the proteins were transferred to PVDF membranes (Millipore, Germany) and blocked with 5% skimmed milk powder for 2 h at room temperature. The membrane was incubated overnight with primary antibody (diluted according to manufacturer’s instructions) at 4 °C. On the second day, the horseradish peroxidase-conjugated secondary antibody diluted at 1:5000 was incubated at room temperature for 1 h. After washing with TBST, the PVDF membrane was detected by a gel automatic exposure imaging system. Image-Pro Plus software was used for gray value analysis of Western blotting.

### Statistical analysis

SPSS 13.0 statistical software was used to analyze the data, the mean ± standard deviation was used to represent the data, and the Wilcoxon test method for comparison of two independent samples of the original data was used to analyze whether the data were significantly different. One-Way ANOVA was used for comparison between groups. The inspection standard shall be as per P < 0.05 indicates a significant difference, * indicates P < 0.05, ** indicates P ≤ 0.01, *** indicates P ≤ 0.001. The experimental data and related statistical data were analyzed by GraphPad Prism 5 software.

## Result

### Prediction of the Que target genes

**Fig. 2:**
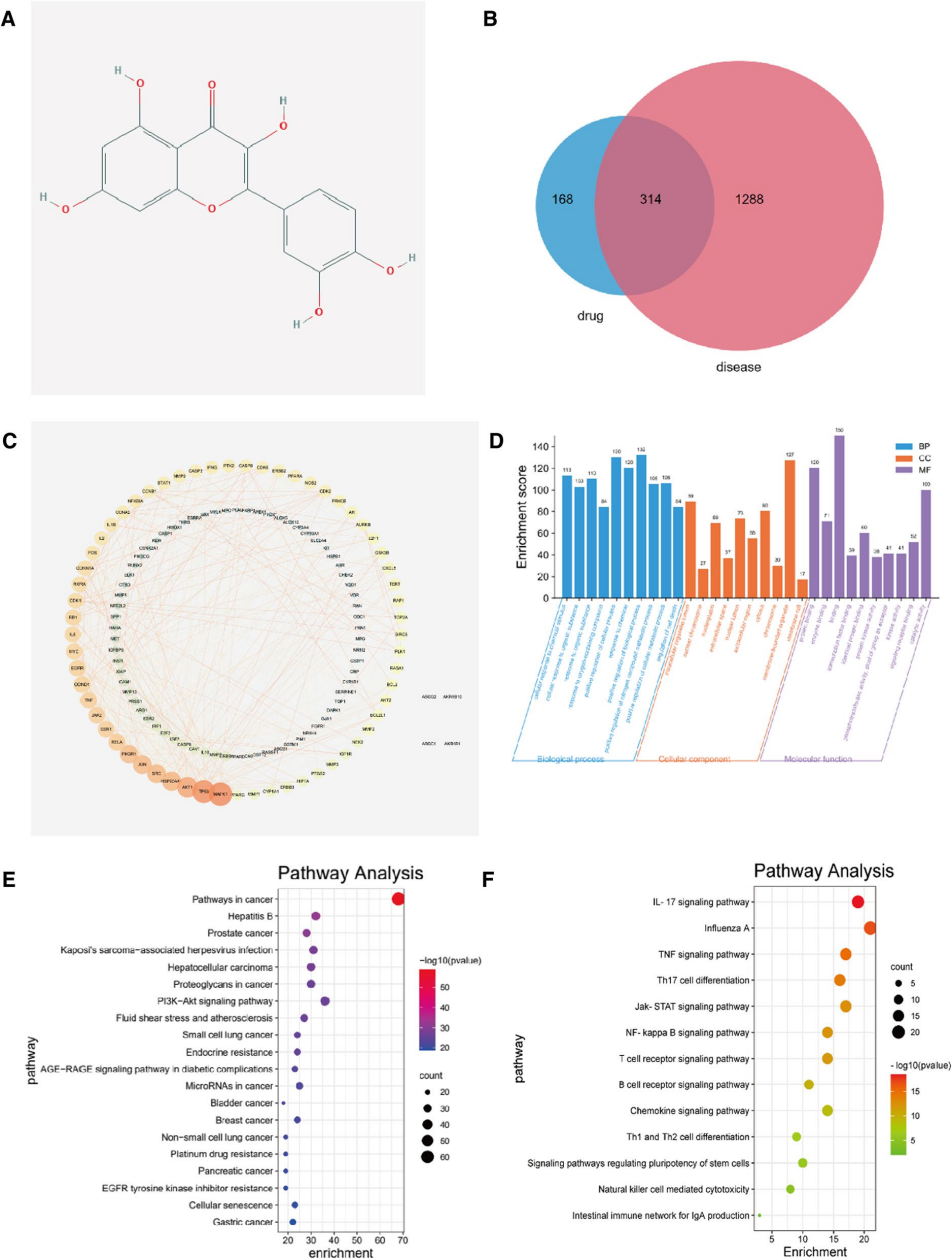
**A** The chemical structure of quercetin (Que). **B** The interaction network of the Que target genes. **C** The PPI network graph of target contains 157 nodes and 589 edges, in which the nodes represent targets and the edges represent the interaction between targets. **D** Gene ontology (GO) enrichment analysis of the Que target genes: the plot of enriched biological processes, the plot of enriched cellular components and the plot of enriched molecular functions. **E** KEGG enrichment analysis of the Que target genes: KEGG annotation of target genes. The number of genes enriched in each KEGG term is shown as the circle size, the p-value shown as different colors. **F** The important genes were mainly distributed in the pathways in cancer. JAT/STAT1 pathways ranked fourth among the pathways screened for immunity to Que

The chemical structure of Que was obtained from the PubChem database, as shown in Fig. 2A. Based on its structure, the PharmMapper database, TCMSP database, and Swiss Target Prediction database were used to predict the potential target genes of Que. The targets obtained from these databases were combined. Duplicates were removed to obtain 482 potential target genes.

### Prediction of precancerous lesions of breast cancer-related que target genes by a systematic pharmacology approach

Information on a total of 1602 disease target genes associated with breast cancer lesions were obtained in the GeneCards database (https://www.genecards.org/) when the keywords "precancerous lesions" were used. Intersecting 482 potential target genes of Que with 1602 disease targets resulted in 314 genes, as shown in Fig. 2B, and these target genes were not only precancerous lesion-related genes but also drug targets. The PPI network graph of the target contained 157 nodes and 589 edges, in which the nodes represent targets and the edges represent the interaction between targets. The PPI data were imported into Cytoscape 3.6.1 software for visualization. As shown in Fig. 2C, the top 20 targets for the degree value of the target were MAPK1, SRC, HRAS, AKT1, HSP90AA1, MAPK8, RHOA, MAPK14, ESR1, EGFR, IGF1, RXRA, JAK2, CASP3, AR, PTK2, IL-2, STAT1 and MMP9.

### Functional enrichment analysis for the Que target genes

To explore the relationship between these 314 potential target genes, GO and KEGG enrichment analyses were performed using the STRING database. A variety of GO enrichment terms were enriched, including 1980 biological processes, 100 cellular components, and 223 molecular functions. We found that biological processes such as cellular process and metabolic process, cellular components such as binding and catalytic activity, and molecular function such as cell and intracellular (Fig. 2D) were enriched, which may be involved in the biological activity of the Que treatment process. In addition, 176 KEGG pathways were enriched (Fig. 2E). Among the major Que-related pathways identified by KEGG, the pathways were mainly associated with the cancer pathway and PI3K-Akt signaling pathway; however, JAK/STAT1 signaling pathway ranked fourth among the pathways screened for immunity to Que (Fig. 2F). It has been suggested that Que may improve the progression of breast cancer from precancerous lesions to breast cancer or improve the prognosis of breast cancer patients through the JAK/STAT1 signaling pathway.

### Cell proliferation experiments in MCF-10AT, MCF-7 and MDA-MB-231 cells

**Fig. 3:**
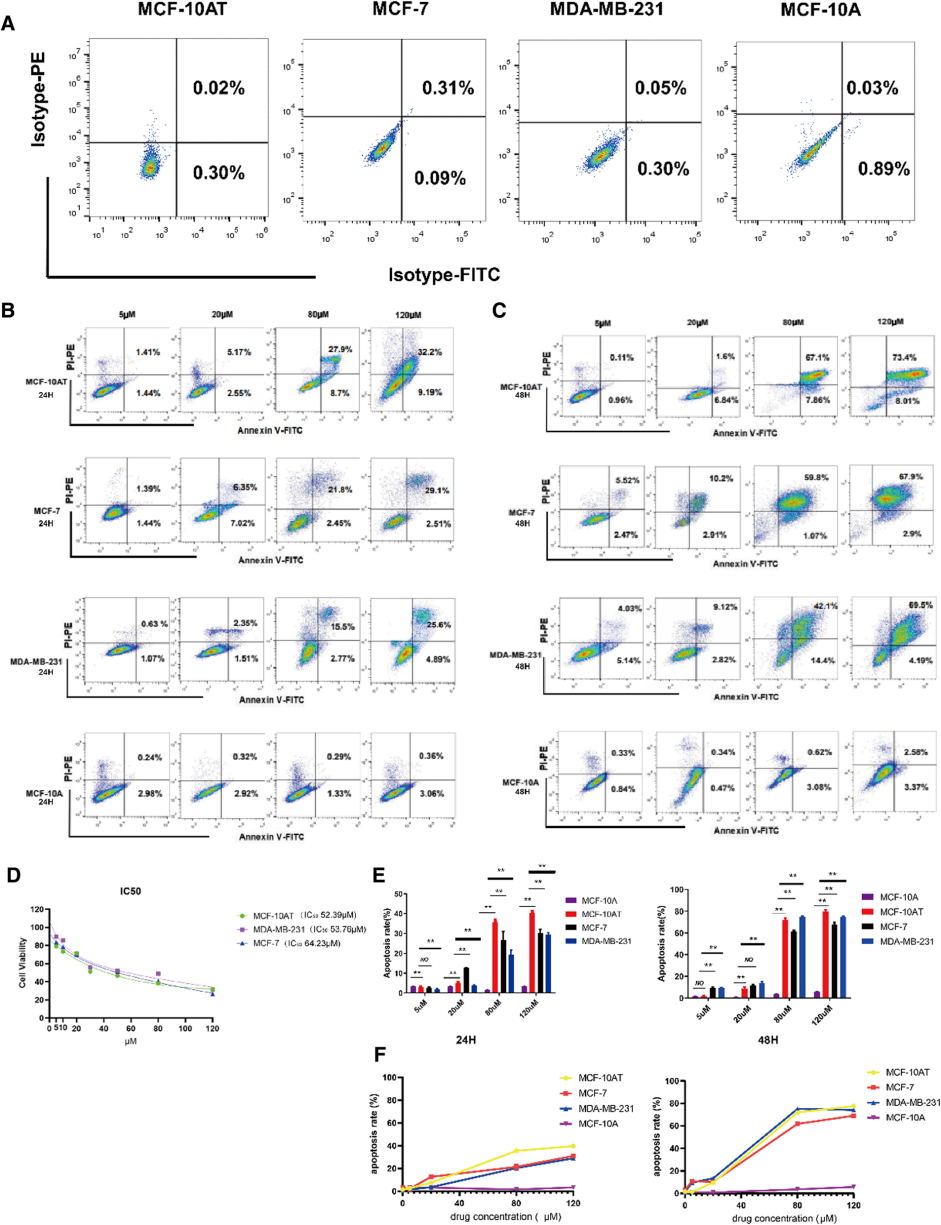
**A**, **B** Flow cytometry was used analysis of apoptotic cells induced by Que using the annexin V/PI double staining assay. Representative dot plots of Annexin V/PI staining. Cells were treated with Que at 5 µM, 20 µM, 80 µM and 120 µM for 24 h and 48 h, respectively. 10,000 cells were analyzed by flow cytometry. The results are expressed as mean ± SD of three independent experiments. p ≤ 0.01(**), as compared to the control. **C** The effects of Que on the proliferation of MCF-10AT, MCF-7 and MDA-MB-231 human breast cancer cells. The cells were treated with Que (5, 10, 20, 40, 60, 80, 100, 120 μM), and the proliferation of the cells was determined by MTT assay. **D** Column bar graph of apoptotic cells. **E** The trend of apoptotic cells

The effects of Que on the proliferation of MCF-10AT, MCF-7 and MDA-MB-231 cells were determined by an MTT assay, and the results are shown in Fig. 3D. The half maximal inhibitory concentration (IC_50_) values of Que on MCF-10AT, MCF-7 and MDA-MB-231 cells were 52.39 µM, 53.76 µM and 64.23 μM, respectively.

### Apoptosis of MCF-10AT, MCF-7 and MDA-MB-231 cells was induced by Que at different concentrations at different time periods

To evaluate the proapoptotic properties of Que on induced cell death, we performed an Annexin-V binding assay and made compensation-related errors (Fig. 3A). We detected the apoptosis of MCF-10A, MCF-10AT, MCF-7, MDA-MB-231 cells treated with Que at 5 μM, 20 μM, 80 μM, and 120 μM at 24 h and 48 h (Fig. 3B and C). First, mammary gland cells were treated with Que for 24 h. We compared MCF-10AT with MCF-10A, MCF-7 and MDA-MB-231 cell lines at a Que concentration of 5 μM. The percentage of the total apoptotic cell population was determined to be 2.85 ± 0.21% and 3.27 ± 0.14%, 2.84 ± 0.21%, and 2.85 ± 0.21% (*P* = *0.006, P* = *0.109,* and *P* = *0.004*, respectively), and the difference was statistically significant. MCF-10AT cells were compared with MCF-10A, MCF-7, MDA-MB-231 cell lines treated with 20 μM Que. The percentage of the apoptotic cell population was 7.60 ± 0.21% and 3.31 ± 0.12%, 12.76 ± 0.37%, and 20.17 ± 2.90% (*P* = *0.004, P* = *0.002,* and *P* = *0.004,* respectively). There was a significant statistical difference. MCF-10AT cells were compared with MCF-10A, MCF-7, and MDA-MB-231 cells treated with 80 μM Que. The percentage of the apoptotic cell population was 35.63 ± 0.60% and 1.64 ± 0.05%, 25.34 ± 5.06%, 29.80 ± 0.98% (*P* = *0.002, P* = *0.004, P* = *0.004,* respectively), which showed statistical significance. MCF-10AT cells were compared with MCF-10A, MCF-7 and MDA-MB-231 cell lines treated with 120 μM Que. The percentage of the apoptotic cell population was 40.25 ± 0.71% and 3.40 ± 0.07%, 29.38 ± 2.50%, and 29.40 ± 2.51% (*P* = *0.002, P* = *0.002,* and *P* = *0.002,* respectively), with statistical significance. Next, MCF-10AT was compared with MCF-10A, MCF-7, MDA-MB-231 cell lines at 5 μM concentration of Que, the percentage of apoptotic cell population was determined as 1.75 ± 0.33% and 1.34 ± 0.15%, 9.63 ± 1.70%, 9.58 ± 0.32% (*P* = *0.240, P* = *0.004,* and *P* = *0.004,* respectively). MCF-10AT was compared with MCF-10A, MCF-7, MDA-MB-231 cell lines at 20 μM concentration of Que and percentage of the apoptotic cell population was determined as 8.44 ± 1.54% and 0.84 ± 0.10%, 11.12 ± 1.38%, 14.23 ± 1.62% (*P* = *0.004, P* = *0.016,* and *P* = *0.004,* respectively) with significant statistical differences. MCF-10AT was compared with MCF-10A, MCF-7 and MDA-MB-231 cell lines at 80 μM Que, and the percentage of apoptotic cell population was determined to be 72.36 ± 1.77% and 3.52 ± 0.14%, 61.95 ± 1.22%, 74.73 ± 0.77% (*P* = *0.004, P* = *0.004,* and *P* = *0.009,* respectively) with statistical significance. MCF-10AT was compared with MCF-10A, MCF-7, MDA-MB-231 cell lines at 120 μM concentration of Que, the percentage of apoptotic cell population was determined to be 79.19 ± 1.76% and 5.52 ± 0.30%, 61.95 ± 1.22%, 74.37 ± 0.80% (*P* = *0.004, P* = *0.004,* and *P* = *0.002,* respectively) with significant statistical differences (Fig. 3E). These results indicate that Que can induce apoptosis in MCF-10AT (precancerous breast cancer cells) and MCF-7, MDA-MB-231 (breast cancer cells) at different periods and at different doses in a time- and concentration-dependent manner. However, Que has a stronger apoptosis effect on MCF-10AT cells with precancerous breast cancer lesions but less or even no effect on MCF-10A cell apoptosis. Apoptosis rates at different concentrations at 24 h and 48 h are shown in Fig. 3F.

### Que promotes γδ T cell amplification

**Fig. 4:**
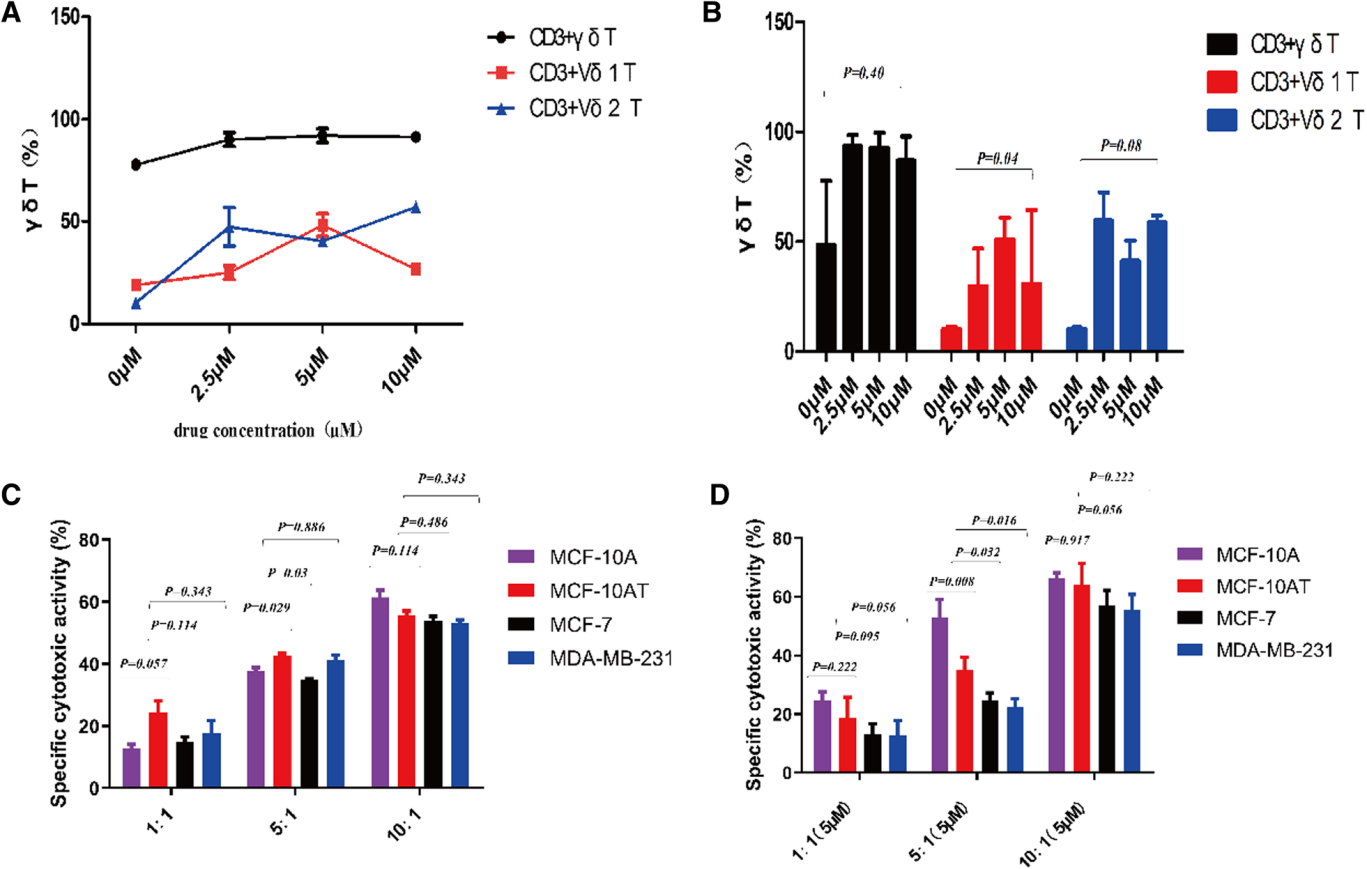
**A**, **B** Purity of γδ T cells during vitro expansion. γδ T cells were treated with Que at 0 µM, 2.5 µM, 5 µM and 10 µM for 10 day or 12 day, respectively. Detecting the frequency of the total lymphocyte cells in CD3 + γδ T cells, Vδ1 + γδ, and Vδ2 + γδ T cell populations. **C** Cytotoxicity of γδ T cells against target cells at 1:1, 5:1 and 10:1 effector to target ratios detected by CFSE staining methods. **D** the effector cell: target cell (E/T) was 1:1, 5:1, 10:1 were treated with Que at 5 µM and for 4 h, synergistic cytotoxic effects on breast cancer cells detected by CFSE staining methods. 20,000 cells were analyzed by flow cytometry

In vitro induction of peripheral blood mononuclear cells from healthy volunteers in complete medium containing TCR γδ monoclonal antibody and cytokine IL-2 was performed. After adding 0 μM, 2.5 μM, 5 μM, and 10 μM Que on Day 3, the cell morphology was observed under a microscope, and the proportion of γδ T cell subsets was determined by flow cytometry after 10–12 days. We found that the number of γδ T cells increased after Que treatment each day in vitro. Flow cytometry analysis showed that γδ T cells could be effectively amplified with more than 60% purity. γδ T cells expanded to 90% at concentrations of 2.5 μM to 5 μM. Vδ2 T cell subsets were dominant at 2.5 μM Que, Vδ1 T cell subsets were dominant at 5 μM Que, and Vδ2 T cell killing subsets were dominant at 10 μM Que (Fig. 4A). Different concentrations of Que had no significant difference in γδ T cells and their Vδ2 T subsets but had a significant difference in Vδ1T cell subsets (*P* = *0.40, P* = *0.08,* and *P* = *0.04,* respectively) (Fig. 4B). These results indicate that γδ T cells have improved killing and immunomodulatory effects.

### Cytotoxicity of healthy human γδ T cells against MCF-10A, MCF-10AT, MCF-7, MDA-MB-231 cell lines

After amplification in vitro, the effector cell: target cell (E/T) ratio was 10:1, the killing rates of γδ T cells against MCF-10A, MCF-10AT, MCF-7, and MDA-MB-231 cells were 61.44 ± 4.70, 55.52 ± 3.10, 53.94 ± 2.74, and 53.28 ± 1.73 (*P* = *0.114, P* = *0.486,* and *P* = *0.343,* respectively) (Fig. 4C), and the trend was 10:1 > 5:1 > 1:1. There was no significant difference between the groups. These results indicate that γδ T cells have a certain killing effect on both precancer and breast cancer cells.

### Que and γδ T cells have synergistic cytotoxic effects on mammary gland cells

To investigate the killing effect of Que and γδ T cells on mammary gland cells, we used 5 μM Que to detect E/T (1:1, 5:1, 10:1) and investigate the specific killing effect. We found that the cell killing rates of MCF-10A cells in E/T (1:1, 5:1, 10:1) were 24.12 ± 4.34, 51.93 ± 6.47, 64.94 ± 3.61. The cytotoxicity rates of MCF-10AT in E/T (1:1, 5:1, 10:1) were 19.38 ± 5.30, 33.45 ± 5.49, 64.96 ± 5.45, and MCF-10A > MCF-10AT (*P* = *0.222, P* = *0.008,* and *P* = *0.917*, respectively). The cell killing rates of MCF-7 at E/T (1:1, 5:1, 10:1) were 13.23 ± 2.68, 24.39 ± 3.13, 55.59 ± 5.98, compared with MCF-10AT, the killing rate of MCF-7 versus MCF-10AT was statistically significant at 5:1 (*P* = *0.095, P* = *0.032,* and *P* = *0.056*, respectively). The cell killing rates of MDA-MB-231 cells at E:T ratios of 1:1, 5:1, and 10:1 were 12.77 ± 3.64, 22.7 ± 1.39, and 59.04 ± 5.67, respectively. Thus, compared with MDA-MB-231 cells, MCF-10AT cells were also statistically significant at a 5:1 ratio (*P* = *0.056, P* = *0.016,* and *P* = *0.222*, respectively). In addition, with the increase in effector cell proportion, the Que concentration was still 5 μM, and MCF-10A > MCF-10AT > MCF-7 > MDA-MB-231, 1:1 < 5:1 < 10:1 (Fig. 4D). These results indicate that Que combined with γδ T cells had a specific killing effect on both precancerous breast cancer cells and breast cancer cells. The strongest killing effect on precancerous breast cancer cells and breast cancer cells was found when the Que concentration was 5 μM and E/T (10:1).

### Effect of Que on IFNγ-R, phospho-JAK2 (p-JAK2), phospho-STAT1 (p-STAT1) and PD-L1 in MCF‐10AT and MCF-7 cell line protein expression

**Fig. 5:**
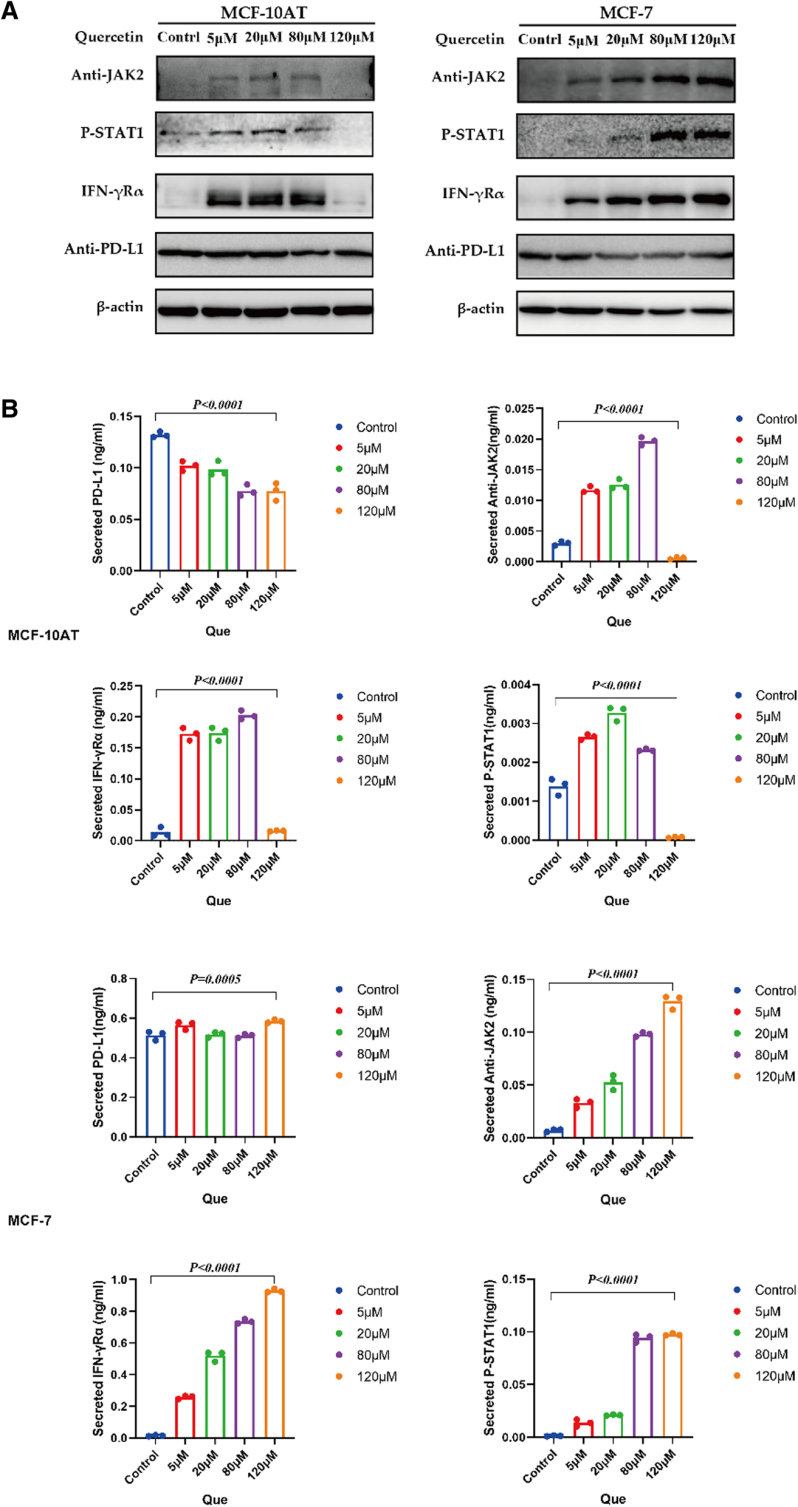
**A** Effect of Que on protein levels of IFNγ-R, p-Jak2, p-STAT1 and PD-L1 in MCF‐10AT and MCF-7 cell lines. **B** Statistical figure of the effect of Que on protein levels of IFNγ-R, p-Jak2, p-STAT1 and PD-L1 in MCF‐10AT and MCF-7 cell lines

Western blotting of IFNγ-R, p-JAK2, p-STAT1 and PD-L1 protein was performed. MCF-10AT and MCF-7 cells were treated with different concentrations of Que at 0 μM, 5 μM, 20 μM, 80 μM and 120 μM for 48 h. Our results showed that when MCF-10AT and MCF-7 cells were treated with Que at 80 μM and 120 μM, respectively, IFNγ-R protein levels and p-JAK2 and p-STAT1 phosphorylation were significantly increased (*P* < *0.0001)*, while PD-L1 protein levels were decreased (*P* < *0.0001, P* = *0.0005*) (Fig. 5A and B).

## Discussion

Breast cancer is a malignant tumor, mostly in females. Since most patients fail to pay attention to precancerous breast lesions, the number of breast cancer patients increases year by year. Precancerous lesions of breast cancer are proliferative lesions with abnormal breast tissue morphology, proliferation or differentiation. After follow-up observation, it is possible that these lesions will develop into cancer. As a stage of tumor development, most of these lesions are in an unstable state and can undergo malignant changes under the continuous action of certain factors. However, if the carcinogenic factors are removed, it is possible to maintain stability or induce degeneration or reversal, and these lesions may return to normal or close to normal. In addition, for precancerous lesions of breast cancer, the main therapeutic drugs are estrogen receptor modulators or aromatase inhibitors, which eventually lead to endocrine disorders and increase the possibility of developing endometrial cancer or uterine fibroids and sarcomas [33]. A study among African women found that breast cancer patients have a better chance of survival if the cancer is detected early or met with immediate clinical intervention. Breast cancer screening (BCS) or breast self-examination (BSE) are key to early detection [34]. Because T cells function to kill tumor cells, they affect the disease progression and prognosis of patients with breast cancer [35]. In-depth study of cellular immune status in patients with precancerous breast cancer will help these patients control the progression of the disease by improving their autoimmune system. For breast cancer patients, their own immune status determines the outcome and prognosis of the disease, which undoubtedly brings new hope to breast cancer patients.

In our previous network pharmacology analysis of Que, we used relevant databases to screen 482 potential genes related to Que and genes related to breast precancerous lesions or breast cancer. Studies have found that these genes are not only related to breast precancerous lesions or breast cancer-related target genes but also Que-related target genes. Then, we uncovered that the biological processes such as cellular process and metabolic process, cellular components such as binding and catalytic activity, and molecular functions such as cell and intracellular were enriched, which may be involved in the biological activity of the Que treatment process. However, in KEGG enrichment analysis, JAK/STAT1 ranked fourth among the pathways that were identified in immunity to Que. Que-related pathways included the JAK/STAT1 pathway, suggesting that Que may improve the progression of breast cancer from precancerous lesions to breast cancer or improve the prognosis of breast cancer patients through the JAK/STAT1 signaling pathway.

The immune system plays an important role in immune defense, immune surveillance and immune homeostasis. When immunity is reduced, aging-damaged mutant cells or invasive pathogens cannot be removed in a timely manner, resulting in the development of tumors or infections. Therefore, improving immune function is important for maintaining health. Que exists widely in nature, and it not only has anti-inflammatory, antitumor, antiplatelet aggregation, endothelial cell protection and antioxidant effects but also has a regulatory effect on immune cells. Studies have shown that Que has a two-way regulatory effect on immune cells. The differentiation of T cell subsets in normal mice was induced, and pathological CD4+ T cells were inhibited to achieve a therapeutic effect on autoimmune myocarditis [36]. In addition, Que also enhanced the immune function of Th2 cells in immunocompromised mice [37]. Other studies have shown that flavonoids can promote immune cell proliferation and enhance immunity [38]. Some scholars found that in an OVA-induced mouse asthma model, Que could regulate the expression of the T-bet and GATA-3 genes and affect the production of Th1/Th2 cytokines, resulting in a decrease in the IL-4 level of Th2 cytokines and an increase in the IFN-γ level of Th1 cytokines [12]. Our current study found that Que induced precancerous breast lesions and apoptosis of breast cancer cells in a time- and concentration-dependent manner. At 24 h, 120 μM Que had the most significant proapoptotic effect on MCF-10AT cells, which was shown as MCF-10AT > MCF-7 > MDA-MB-231 > MCF10A. At 48 h, 80 μM Que had an apoptosis effect on breast cancer precancerous cells and breast cancer cells. At the same time, it was consistent with the IC_50_ value of Que. In addition to a pro-apoptotic effect, Que had a regulatory effect on immune cells and had a promoting effect on the proliferation of γδ T cells and their subsets. When the concentration of Que was 5 μM, the Vδ1 T cell regulatory subsets of γδ T cells were dominant and played an immunomodulatory role. However, with increasing concentration, the killing subpopulation of Vδ2 T cells gradually became dominant. The results showed that Que could promote the differentiation of γδ T cells into Vδ2 T cells and strengthen their antitumor ability. This suggests that Que has a stimulating effect on the immune system.

γδ T cells have antiviral and antitumor effects, with promising transformation and application prospects in immunotherapy. However, due to the obvious heterogeneity of γδ T cells in patients, different subsets may have different functions. Previous studies have shown that γδ T cells can play the role of Th1 (type 1 helper T lymphocytes) by producing IL-2 and IFN-γ when intracellular bacteria are infected. When extracellular parasites and other infections occur, interleukin-10 (IL-10) and interleukin-4 (IL-4) are produced and play a role in Th2 (type 2 helper T lymphocytes) regulation [39]. With further research on γδ T cells, γδ T cells were found to be related to the occurrence and development of diseases. Noguchi et al. found that the number of γδ T cells in patients with solid tumors such as breast cancer, lung cancer and gastric cancer was significantly lower than that in healthy controls because chemotherapy inhibited the proliferation of γδ T cells [22]. In this study, we found that when the ratio of γδ T cells to tumor cells was 10:1, γδ T cells had the strongest killing effect on tumor cells. Then, we cocultured tumor cells with γδ T cells in the absence or presence of Que and found that when the concentration of Que was 5 μM and E/T was 10:1, the killing effect on MCF-10A, MCF-10AT, MCF-7 and MDA-MB-231 cells reached a maximum. These results indicated that Que and γδ T cells could further enhance the killing effect on tumor cells and that Que and γδ T cells may have synergistic effects. These results show that Que has a positive regulatory effect on cellular immune function. Therefore, a diet including hawthorn, honey, juice and other foods, which are rich in Que in daily life, would be helpful to regulate immune function. However, due to the complex biological characteristics of γδ T cell subsets, it is necessary to conduct more in-depth research on their functional subsets to provide more data for individualized immunotherapy and a comprehensive understanding of the cellular immune status of precancerous breast lesions and breast cancer patients.

The JAK/STAT1 pathway is involved in almost the entire immune regulation process, including immune surveillance and immune escape. STAT1 promotes cell apoptosis, inhibits cell growth and differentiation and plays an important role in inhibiting tumorigenesis and tumor development by regulating the interferon (IFN) system [40]. STAT1-dependent IDO overexpression blocked the activation of T cells, which was related to an increase in PD-L1 expression in high-level triple-negative breast cancer (TNBC) cells [41]. In many cancer diseases, STAT1 overexpression also promotes tumor cell survival and immune depletion by prolonging IFN-γ signaling. Some cytokines act on the receptor IFNγ-R on tumor cells. After phosphorylation by JAK1/2, they promote tumor immunogenicity by upregulating MHC and immune checkpoint proteins (such as PD-L1 and B7-1) through STAT1 signaling of transcription factors [42]. Studies have shown that the pathogenesis of breast cancer is closely related to a significant increase in the expression levels of inflammatory factors such as TNF-α and IL-6 in TNBC. Furthermore, it is closely related to the increase in PD-L1 [31]. The expression of these cytokines may be regulated by IFNγ-R, nuclear transcription factor-STAT1 and JAK1/2. Our results showed that after treatment of MCF-10AT and MCF-7 cells with 80 μM or 120 μM Que for 48 h, the levels of IFNγ-R, p-JAK2 and p-STAT1 increased, and the level of PD-L1 decreased. These results indicate that Que further increases the phosphorylation of JAK2 on the JAK/STAT1 pathway by activating IFNγ-R on tumor cells and increases the nuclear localization of p-STAT1, thus reducing the expression of PD-L1 on the precancerous cells of breast cancer. Que further reduced the binding of PD-L1 and PD-1 on the T cell receptor, avoiding weakening of antitumor immunity by T cell activation signals. By doing so, immune cells can play a normal immune role. Thus, the JAK-STAT1 pathway has become an attractive therapeutic target in precancerous lesions of breast cancer as well as in breast cancer development and progression.

## Conclusions

We demonstrated for the first time the proapoptotic effect of Que on different breast cancer cells and explored the mechanisms by which Que influences immunity by affecting the JAK/STAT1 signaling pathway. Notably, Que promotes the proliferation of Vδ2 T subsets of γδ T cells, thus enhancing the killing effect on breast cancer cells. This finding suggests that Que and γδ T cells play a synergistic role in killing tumor cells and cellular immune regulation. Que, as a natural flavonoid, should be widely used in clinical practice due to its antitumor and immune regulation functions. Que may be a potential drug for the prevention of precancerous breast cancer and adjuvant treatment of breast cancer.

## Data Availability

The data sets analyzed in the present study are publicly available data from the PubChem database, PharmMapper database, TCMSP database, Swiss Target Prediction database and GeneCards database. The original contributions presented in the study are included in the article. Further inquiries can be directed to the corresponding authors.

## Abbreviations

BCS: Breast cancer screening
BSE: Breast self-examination
CT26: Colon 26
Que: Quercetin
IL-2: Interleukin-2
IL-10: Interleukin-10
IL-4: Interleukin-4
MDR: Multi-drug resistance
MMPs: Matrix metallo-proteinases
PD-1: Programmed death receptor 1
PD-L1: Programmed cell death 1 ligand 1
STAT1: Signal transducer and activator of transcription 3
TIMPs: Tissue inhibitor of metalloproteinases
Th1: Type 1 helper T lymphocytes
Th2: Type 2 helper T lymphocytes
TNBC: Triple negative breast cancer
WHO: World Health Organization
